# Risk of death among those awaiting treatment for HIV infection in Zimbabwe: adolescents are at particular risk

**DOI:** 10.7448/IAS.18.1.19247

**Published:** 2015-02-23

**Authors:** Amir Shroufi, Wedu Ndebele, Mary Nyathi, Hilary Gunguwo, Mark Dixon, Jean F Saint-Sauveur, Fabian Taziwa, Mari C Viñoles, Rashida A Ferrand

**Affiliations:** 1 Médecins Sans Frontières, Harare, Zimbabwe; 2 Mpilo OI/ART Clinic, Bulawayo, Zimbabwe; 3 Faculty of Medicine, National University of Science and Technology, Bulawayo, Zimbabwe; 4 Department of Clinical Research, London School of Hygiene and Tropical Medicine, London, UK

**Keywords:** HIV/AIDS, ART, adolescent, pre-ART, Africa

## Abstract

**Introduction:**

Mortality among HIV-positive adults awaiting antiretroviral therapy (ART) has previously been found to be high. Here, we compare adolescent pre-ART mortality to that of adults in a public sector HIV care programme in Bulawayo, Zimbabwe.

**Methods:**

In this retrospective cohort study, we compared adolescent pre-ART outcomes with those of adults enrolled for HIV care in the same clinic. Adolescents were defined as those aged 10–19 at the time of registration. Comparisons of means and proportions were carried out using two-tailed sample *t*-tests and chi-square tests respectively, for normally distributed data, and the Mann–Whitney *U*-tests for non-normally distributed data. Loss to follow-up (LTFU) was defined as missing a scheduled appointment by three or more months.

**Results:**

Between 2004 and 2010, 1382 of 1628 adolescents and 7557 of 11,106 adults who registered for HIV care met the eligibility criteria for ART. Adolescents registered at a more advanced disease stage than did adults (83% vs. 73% WHO stage III/IV, respectively, *p<*0.001), and the median time to ART initiation was longer for adolescents than for adults [21 (10–55) days vs. 15 (7–42) days, *p<*0.001]. Among the 138 adolescents and 942 adults who never commenced ART, 39 (28%) of adolescents and 135 (14%) of adults died, the remainder being LTFU. Mortality among treatment-eligible adolescents awaiting ART was significantly higher than among adults (3% vs. 1.8%, respectively, *p*=0.004).

**Conclusions:**

Adolescents present to ART services at a later clinical stage than adults and are at an increased risk of death prior to commencing ART. Improved and innovative HIV case-finding approaches and emphasis on prompt ART initiation in adolescents are urgently needed. Following registration, defaulter tracing should be used, whether or not ART has been commenced.

## Introduction

There has been a rapid and substantial scale up of antiretroviral therapy (ART) provision in sub-Saharan Africa over the past decade which has been accompanied by substantially increased survival of HIV-positive individuals [1]. There has been much focus in HIV programmes on outcomes among those who start ART, but HIV-positive individuals awaiting initiation of ART have been largely overlooked [2,3]. Management of HIV-positive individuals in the pre-ART period should include regular clinical and CD4 count monitoring and provision of cotrimoxazole prophylaxis. Mortality and loss to follow-up (LTFU) in this period can be high but individuals defaulting from services at the pre-ART stage are often not followed up, unlike among those missing clinic appointments after commencing ART [3–7] .

It has been recognized in recent years that many children with vertically acquired HIV infection in countries with generalized epidemics are entering adolescence undiagnosed [8]. Delayed presentation of these children to healthcare services has been cited as one reason why mortality is generally the highest in the first few months following initiation [9]. Delayed diagnosis is also associated with chronic complications such as growth failure, neurocognitive impairment, and lung and cardiac disease [10,11]. Further, avoidable delays between registration and the administration of ART are likely to be critically important [5,12,13]. In most cases, there is a prerequisite period of counselling before ART is initiated which can introduce an additional delay in commencing ART, particularly where coordination within clinical services is lacking [5]. Older children and adolescents may be at higher risk of mortality because of the longer duration of having lived with untreated HIV infection and are less able to afford further delays before commencing treatment [14,15].

We conducted a retrospective cohort study to investigate retention in care and mortality in the pre-ART period, as well as factors associated with early programme mortality among adolescents and adults registered in a large public sector HIV care programme in Zimbabwe.

## Methods

### Study setting and participants

Bulawayo, Zimbabwe's second most populous city is located in the southwest of the country and is home to 680,000 people (Population Statistics Office December 2010). The Mpilo ART Clinic in Bulawayo was one of the first public sector HIV care services to be established in Zimbabwe and has provided ART since 2004, accumulating one of the nation's largest patient cohorts. Mpilo Clinic was the only provider of paediatric and adolescent ART in Bulawayo during the study period (2004 to 2010). Patients enrolling for care had usually undergone HIV testing at an inpatient or outpatient department of Mpilo hospital. HIV testing was also available in primary care clinics within Bulawayo, and in a variety of freestanding voluntary counselling and testing (VCT) facilities. Throughout the study period, HIV care at Mpilo was provided by the public sector in partnership with Médecins Sans Frontières (MSF) who supported staffing, service development, and monitoring and evaluation. Volunteers were trained in defaulter tracing which aimed to bring defaulters back into care with those who carried out this work receiving incentives from a partner organization. Both telephone follow-up and home visits were used, with all those missing a visit by two or more months being eligible for defaulter tracing. Defaulter tracing focussed mainly on those who had commenced ART rather than pre-ART patients.

Eligible patients were adults and adolescents who enrolled for HIV care between April 2004 and December 2010 at the clinic. Age was defined from the date of an individual's first visit to the clinic. In line with the WHO definition, an adolescent was defined as someone aged ≥10 and <19. Analyses included both treatment naïve patients and those with a history of having been on ART prior to enrolment. The pre-ART period was defined as the period between registration and initiation of ART. Individuals who missed a scheduled appointment by three months or more were classified as LTFU. In line with the national guidelines, ART eligibility was met if an individual had a CD4 count less than 200 cells/mm^3^ and/or a WHO Stage III or IV illness.

### Data collection

Since the clinic's inception in 2004, details of all patients have been systematically entered into an Access database using software called FUCHIA (Epicentre, Paris, France). This database is routinely updated during each patient follow-up visit and includes details of anthropometric measures, CD4 counts, drug regimen details and treatment outcomes. This database provided data on age, sex, date of commencing ART, drug regimen, dates of clinic attendance, WHO clinical stage, CD4 count and other laboratory recordings. LTFU status was updated on an ongoing basis, allowing reclassification of patients who returned to the clinic after a period of absence. Mortality data were obtained through systematic tracing of defaulters, as well as from community reporting and an ongoing death register review. The last date for data collection was 31 December 2010.

### Statistical analysis of data

All statistical analyses were carried out using STATA version 10 (Stata-Corp, TX, USA). Comparisons of means and proportions were carried out using two-tailed sample *t*-tests and chi-square tests respectively for normally distributed data. The Mann-Whitney U-test was used for the analysis of non-normally distributed data. To assess the impact of delayed initiation on mortality, a metric we refer to as “early programme mortality” was constructed, defined as any death (pre- or post-ART) occurring within 12 months of registration among patients eligible for ART at registration. We used linear regression to analyze secular trends in early programme mortality over time; the dependent variable being early programme mortality and the independent variable being year of ART initiation. We performed a survival analysis comparing time to event between groups using the log rank test to establish if observed differences were statistically significant.

### Ethical considerations

Only routine data, collected for patient management and programme monitoring were used. Individual consent to use clinical data was not obtained. Patient confidentiality was preserved by not extracting personal information which could directly identify patients and records were anonymized to maintain patient confidentiality. Ethical approval for this study was obtained from the Mpilo Hospital Ethical Review Board, who was informed that individual consent was not obtained for reasons of feasibility as outlined above.

## Results

Demographic and registration information was missing for 167 records. During the study period, 1973 adolescents and 11,106 adults completed initial registration at the clinic, of whom 345 adolescents and 2056 adults had insufficient data at registration to determine ART eligibility; these records were excluded from analysis. This equated to 3519 person-years (pyrs) of follow-up in adolescents and 20,458 in adults. There was no difference in the hazard of mortality following registration, comparing those excluded from analysis with those included (*p*=0.37, log rank).

**Figure 1:**
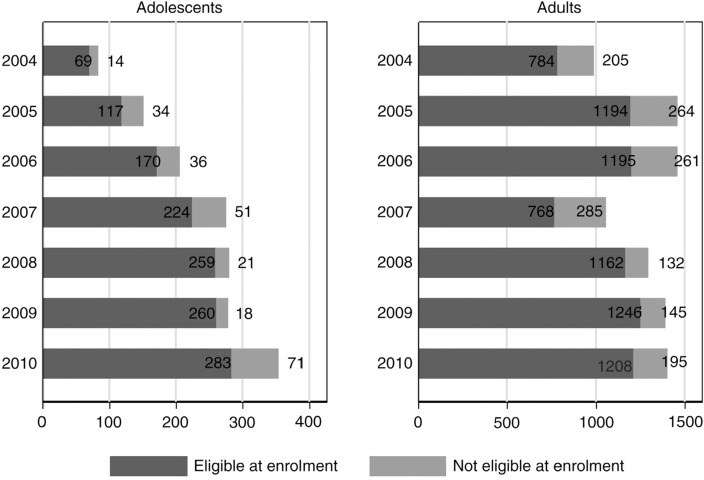
Numbers of adolescents and adults enrolling at Mpilo Clinic between 2004 and 2010 according to ART eligibility at time of enrolment.

Of the 1628 adolescents and 9050 adults included in the analysis, 1382 (85%) adolescents and 7557 (84%) adults satisfied ART eligibility criteria at enrolment. Between 2004 and 2010, the number of treatment-eligible adolescents registering for HIV care increased four-fold, compared to a less than two-fold increase in the corresponding number of adult registrations (Figure 1).

### Characteristics at registration into care

Among adults who registered for care, more than two-thirds were female, but there was no difference by sex among adolescents registering for HIV care. The median age at registration was 13.1 [interquartile range (IQR) 11.4–15.2] years for adolescents and 36.6 (IQR 31.0–43.6) years for adults (Table 1). The vast majority (>95%) of adolescents were assessed by clinicians to have been vertically infected based on history of parental death or HIV status, self-report of sexual debut, or blood transfusions and clinical history; although it is possible that we may have underestimated the proportion who were horizontally infected due to social desirability bias affecting responses to questions related to mode of transmission.

**Table 1:** Baseline characteristics of patients at registration at the Mpilo Clinic

	Total	Adults	Adolescents	*p*
Total number of registered patients	10,678	9050	1628	–
Median age – years (IQR)	34.7 (27.0–41.9)	36.6 (31.0–43.6)	13.1 (11.4–15.2)	–
Male	35% (3684/10,678)	32% (2916/9050)	47% (768/1628)	<0.001
Previous ARV history	14% (1441/10,678)	15% (1365/9050)	3% (41/1623)	<0.001
VCT/self-referral	16% (1696/10,645)	18% (1642/9025)	3% (54/1620)	<0.001
Percentage eligible to start ART	84% (8939/10,678)	84% (7557/9050)	85% (1382/1628)	0.164
WHO Stage III/IV HIV disease	74% (7526/10,161)	73% (6316/8707)	83% (1210/1457)	<0.001
Median CD4 count, cells/mm^3^ (IQR)	167 (75–285) (*n*=6445)	162 (75–275) (*n=*5535)	210 (85–370) (*n=*910)	<0.001
CD4 count <200cells/mm^3^	58% (3736/6445)	60% (3292/5535)	49% (444/910)	<0.001
Haemoglobin <11g/dl	85% (2684/3144)	40% (1072/2684)	47% (216/460)	0.005
Pulmonary tuberculosis	10% (1011/10,678)	9% (836/9050)	11% (175/1628)	0.055
Severe pneumonia	3% (282/10,678)	2% (220/9050)	4% (62/1628)	<0.001

Most adolescents underwent HIV testing following an HIV-associated illness, with far fewer accessing HIV testing through VCT services compared to adults (3.3% vs. 18.2%, *p<*0.001) (Table 1). Adolescents presented at a more advanced WHO disease stage than adults, with 83% of adolescents presenting with WHO Stage III or IV disease, compared to 73% of adults (*p<*0.001). A history of pulmonary TB, bacterial pneumonia and anaemia was also significantly more common among adolescents. However, adolescents had a higher median CD4 count at the time of registration compared to adults (210 vs. 162 cells/mm^3^, *p<*0.001). Adults were more likely to have had prior exposure to ART than adolescents (15.7% vs. 5.4%, *p<*0.001).

### Pre-ART LTFU and mortality

Among those eligible for ART, 138 adolescents and 942 adults had no record of ever initiating treatment. Of these, 39 (28%) adolescents and 135 (14%) adults were known to have died before starting ART, and the remainder did not start treatment because they were LTFU. The mortality rate among treatment-eligible adolescents who did not commence ART was 29.0 per 100 pyrs [95% confidence interval (CI): 21.1–39.6], significantly higher than that among adults who did not commence treatment (8.0 per 100 pyrs; 95% CI: 6.8–9.0), (*p<*0.005). Following registration, treatment-eligible adolescents waited significantly longer than adults to commence ART, with the median time to ART initiation being 21 (10–55) days in adolescents vs. 15 (7–42) days in adults, *p<*0.001).

Early programme mortality among adolescents and adults declined over time (univariate linear regression of year of registration against early programme mortality, *p*=0.006 and *p=*0.048, respectively) but fell more sharply in adults, and mortality rates remained generally higher among adolescents than in adults each calendar year (Table 2).

**Table 2:** Secular trends in eligibility at clinic registration, time between eligibility and ART initiation and early programme mortality in a) adolescents and b) adults registering for HIV care at Mpilo Clinic from 2004–2010

Year	No. of ART-eligible individuals	*N* (%) with WHO stage IV disease^a^	*N* (%) who never initiated ART	Median (IQR) time in days between registration and ART initiation	*N* (%) who died within one year of first visit (early programme mortality)
**a) Adolescents**					
2004	784	372/782 (48%)	183 (23%)	−0 (0–0)*	137 (18%)
2005	1194	421/1186 (36%)	219 (18%)	65 (35–90)	123 (10%)
2006	1195	269/1182 (23%)	110 (9%)	21 (13–29)*	59 (5%)*
2007	768	168/680 (25%)	67 (9%)	19 (14–28)	43 (6%)
2008	1162	403/1130 (36%)	114 (10%)	14 (14–28)*	22 (2%)*
2009	1246	398/1225 (33%)	100 (8%)	14 (8–24)*	32 (3%)*
2010	1208	213/1179 (18%)	149 (12%)	14 (4–17)*	12 (1%)*
**b) Adults**					
2004	69	20/64 (31%)	9 (13%)	21 (0–49)*	16 (23%)
2005	117	44/104 (42%)	13 (11%)	45 (21.5–87.5)	13 (11%)
2006	170	41/154 (27%)	19 (11%)	23 (14–63)*	15 (9%)*
2007	224	57/204 (28%)	28 (13%)	21 (13–40.5)	13 (6%)
2008	259	86/251 (34%)	31 (12%)	19.5 (11–49)*	12 (5%)*
2009	260	110/258 (43%)	15 (6%)	14 (9–42)*	13 (5%)*
2010	283	109/272 (40%)	23 (8%)	14 (7–24)*	21 (7%)*

a Total number of eligible individuals who had a WHO staging recorded at time of first visit shown.

* Statistically significant (*p*<0.05) difference between adults and adolescents.

## Discussion

The main finding of this study was that HIV-positive adolescents are at a significantly higher risk of death than adults while awaiting commencement of ART. This is likely explained by the fact that adolescents registered at a more advanced stage of HIV infection than did adults. The vast majority of adolescents had evidence supporting vertical infection. Most had been diagnosed recently and had no history of prior ART use. The fact that they were only diagnosed in adolescence implies a delay in diagnosis of over a decade and highlights the inadequacies of current HIV case-finding strategies for children. Over the study period, VCT services were scaled up, enabling earlier diagnosis of HIV infection. However, these services were available almost exclusively for adults, with testing amongst adolescents primarily being carried out in healthcare facilities and only after presentation with an illness requiring hospitalization [14]. There was an additional delay in commencing ART following registration, and this period was significantly longer for adolescents than for adults, which may reflect underpreparedness to initiate treatment as well as factors such as reduced independent access to transport among adolescents. Interestingly, adolescents had higher WHO disease stage than adults but higher CD4 counts than adults at time of registration. Current CD4 count thresholds for starting ART among adolescents with vertically acquired HIV infection may warrant being higher than those for adults [15,16].

Approximately one in ten ART-eligible individuals did not commence ART and were LTFU. Although we cannot establish the underlying reasons for this in our cohort, a study in Malawi within which similar numbers of eligible patients failed to commence ART, found 58% of such patients to have died [3,17]. Patients who do not die may return at a later stage with more advanced disease whereas some may have re-registered for HIV care elsewhere. Whilst most defaulter tracing focuses on those who have initiated ART, these findings underscore the importance of active follow-up for patients in the pre-ART period. The importance of the pre-ART period is highlighted here, as elsewhere, by the finding that about 21% of all programmatic deaths in our cohort occurred prior to the initiation of ART [18–20]. As has been pointed out by others, if pre-ART deaths are not included in routine programme reporting, then overall programme outcomes may appear misleadingly favourable [3,5]. Principally this is because those with more advanced disease are more likely to die in the pre-ART period, with those commencing ART having a relative survival advantage. Thus, programmes with many pre-ART deaths may record fewer early on-ART deaths.

In an effort to assess the full programmatic impact of delayed initiation, we examined mortality among ART-eligible individuals within 12 months of registration, whether or not they commenced ART (which we refer to as early programme mortality). We believe that this provides a more useful indicator of how well a programme manages patients from the time they are registered, because it incorporates pre-ART deaths as well as deaths among those newly commenced on ART, which may be partly attributed to pre-ART delays. It has been proposed that the pre-ART period be analyzed at three stages [7].

Stage 1 is the period from a positive HIV test to receiving the results of an initial CD4 count and being referred to either pre-ART care or ART. Stage 2 is the pre-ART period, spanning the period from enrolment for HIV care until ART eligibility. Finally, Stage 3 is the time between an individual being identified as eligible for ART and the commencement of treatment. In this study, we have focussed on the third stage. Overall LTFU and mortality across all three pre-ART stages is likely to be considerably higher than is shown here.

To our knowledge, this is the first study to investigate outcomes among adolescents awaiting ART. The large sample size, long duration of follow-up, and the comprehensive patient data collected throughout the study period are strengths of this study. The limitations of this study are that we do not have data on the reasons why individuals defaulted from services prior to commencing ART. We cannot therefore establish the relative contribution of death, relocation or other factors (such as individual unwillingness to start). As many of those LTFU before starting ART may have died, the death rates among those not commencing ART are likely to be underestimated [3].

In summary, our study showed that adolescents present to HIV care services at a later clinical stage than adults and are at higher risk of death than adults while awaiting initiation of ART. Improved and innovative HIV case-finding approaches and emphasis on prompt ART initiation in adolescents are urgently needed. Adolescents are poorly served by existing case-finding services such as VCT, a situation likely to contribute to their late presentation to services, in turn contributing to higher death rates among those awaiting ART. Earlier identification of HIV-positive adolescents will require development of new interventions given that current approaches largely fail older children and adolescents. This must be accompanied by linkage to care and timely initiation of ART for treatment-eligible adolescents. The study highlights the importance of the pre-ART period as a substantial contributor to overall programme deaths. Social support models, such as peer support clubs have generally focussed on those who have initiated ART and in future should aim to include individuals who are not yet eligible for ART. Pre-ART outcomes should be incorporated in routine HIV programme reporting, and defaulter tracing activities should also include those who have not commenced ART.
